# Testing patient-informed approaches for visually depicting the hemoglobin A1c value to patients with poorly controlled diabetes: a randomized, controlled trial

**DOI:** 10.1186/s12913-020-5035-8

**Published:** 2020-03-06

**Authors:** Anjali Gopalan, Leah Suttner, Andrea B. Troxel, Kevin McDonough, Marilyn M. Schapira

**Affiliations:** 1 Division of Research, Kaiser Permanente Northern California, 2000 Broadway, Oakland, CA 94612 USA; 2 Department of Biostatistics and Epidemiology, Perelman School of Medicine, University of Pennsylvania, 423 Guardian Drive, Philadelphia, PA 19104 USA; 3 Department of Population Health, NYU Langone Health, 180 Madison Avenue, New York, NY 10016 USA; 4 School of Arts and Sciences, University of Pennsylvania, 120 Claudia Cohen Hall, 249 South 36th Street, Philadelphia, PA 19104 USA; 5 Division of General Internal Medicine, Perelman School of Medicine at the University of Pennsylvania, 3400 Civic Center Boulevard, Philadelphia, PA 19104 USA; 6 The Corporal Michael J. Crescenz VA Medical Center, 3900 Woodland Avenue, Philadelphia, PA 19104 USA

**Keywords:** Diabetes, Hemoglobin A1c, Qualitative research, Patient-provider communication, Patient portal

## Abstract

**Background:**

Patients’ understanding of the hemoglobin A1c (HbA1c) has been linked to better diabetes care outcomes (glycemic control, self-care)**.** This is concerning given low documented rates of HbA1c understanding. In this non-blinded, randomized trial, we compared two formats for communicating the HbA1c, selected based on input from people with diabetes, to standard presentation to assess their impact on participants’ glycemic control and diabetes-related perceptions.

**Methods:**

To design the tested formats, we interviewed 25 patients with diabetes and reviewed a range of possible formats, including color-based scales and graphs. The interviews were recorded, transcribed, and subjected to thematic analysis. Synthesizing interviewees’ feedback, we selected two formats, one using a combination of words and colors (Words) and one using a color-coded graph (Graph), for further evaluation. We then randomized adults with poorly controlled diabetes to receive mailed information on their current diabetes control in one of three ways: 1) standard lab report (control), 2) Words format, or 3) Graph format. The primary outcome was HbA1c change at 6 months. Also examined were changes in participants’ diabetes-related perceptions and choice of participation incentive.

**Results:**

Of the 234 enrolled participants, 76.9% were Black, and their median baseline HbA1c was 9.1% (interquartile range 8.4–10.4). There were no between-arm differences in HbA1c change (− 1.04% [SD 2.2] Control vs. -0.59% [SD 2.0] Words vs. -0.54% [SD 2.1] Graph, *p* > 0.05 for all comparisons). Participants in the Words arm had an increase in the accuracy of their perceptions of diabetes seriousness (*p* = 0.04) and in the number of participants reporting a diabetes management goal (*p* = 0.01).

**Conclusions:**

The two patient-informed communication formats did not differentially impact glycemic control among adults with inadequately controlled diabetes. However, a significant proportion of participants in the Words arm had an increase in the accuracy of their perception of diabetes seriousness, a potential mediating factor in positive diabetes-related behavioral changes. With increasing use of patient-facing online portals, thoughtfully designed approaches for visually communicating essential, but poorly understood, information like the HbA1c to patients have the potential to facilitate interpretation and support self-management.

**Clinical trial registration:**

Prospectively registered as NCT01886170.

## Background

Among adults with diabetes, a better understanding of disease management targets, specifically the hemoglobin A1c (HbA1c), has been associated with better care outcomes, including glycemic control and higher confidence with self-care [1, 2]. However, for many patients, the HbA1c value may be confusing as it is expressed as a percentage, the name has no apparent connection with diabetes, and and it is assessed using a non-intuitive scale (with a goal of 7% or less for most patients). In fact, studies estimate that the minority of patients with diabetes can accurately describe the meaning of the HbA1c value and recall their most recent value [1, 3, 4]. A 2014 study demonstrated that patients with low health literacy had difficulty discerning whether an HbA1c value was in or out of goal range even when the range was presented [5]. Past work has demonstrated that providing diabetes-related feedback to patients in thoughtfully constructed ways (i.e., easy to understand, personally relevant) can promote positive health behavior changes [6]. Building on this literature, we contend that there are ways of presenting the HbA1c value that can increase its comprehensibility and meaningfulness and, potentially, its impact on care outcomes for patients with diabetes.

Examples from other health contexts support this contention. A 2008 study examined smoking cessation rates among individuals who were given feedback on their lung function using either the standard measure, forced expiratory volume, or a calculated “lung age” [7]. Patients given their “lung age” had significantly higher rates of smoking cessation at the end of the study. Additionally, a 2014 study by Thorndike et al. demonstrated the impact of a color-based nutritional labeling system on increasing healthier food purchases [8].

In an initial effort to apply this approach to the HbA1c, in a past study, we compared the effect of translating the HbA1c into one of two alternative formats - letter grades (A through F), and emoticons (sad to smiling) - on participants’ glycemic control and assessments of their current diabetes control compared to a standard presentation of the value [9]. While no between-arm differences were seen in these examined outcomes, a major limitation of this study was that the two tested formats were selected without any input or insights from patients with diabetes.

In this mixed method study, we attempted to address this limitation. We collected feedback on a range of visual formats for conveying information on glycemic control from patients with diabetes. This feedback then informed the design of two final visual formats that we evaluated in a three-arm randomized, controlled trial (RCT) of patients with inadequately controlled diabetes. We hypothesized that those participants randomized to receive one of the two novel HbA1c presentation formats would demonstrate improved glycemic control compared to those receiving the standard HbA1c format. This hypothesis was grounded in several established models of health behavior change in which an individual’s awareness and assessment of their current disease status contribute to positive behavior changes and improved outcomes (e.g., improved glycemic control). Examples include the role of “perceived disease severity and susceptibility” in the Health Belief Model (HBM), the role of “consciousness raising” in the shift from pre-contemplation to contemplation in the Transtheoretical Model of Health Behavior change, and the “informed, activated patient” in Wagner’s Chronic Care Model [10–12].

## Methods

### Intervention development

To develop the tested interventions, we interviewed 25 patients with diabetes (see Supplemental Table 1 for characteristics of interviewees) to elicit their feedback on nine visual formats whose design was primarily grounded in the HBM and informed by communication strategies in other contexts (e.g., forest fire risk meters, fundraising trackers, and popular rating sites like Yelp) (Supplemental Figure 1, Supplemental Table 2) [13–21]. Each participant participated in one semi-structured interview that lasted between 35 and 70 min and was conducted in a private area by a research assistant (KM). Interviews were audio-recorded and transcribed. During the interviews, we collected demographic information, diabetes duration, and self-reported history of diabetes-related complications. Interview participants each reviewed between four and seven of the formats, presented in a random order. Two quantitative measures were also collected from interview participants: 1) accuracy in ranking the level of glycemic control depicted (from best to worst) and 2) ratings of format clarity (five-point Likert scale ranging from “Very Confusing” to “Very Clear”).

For each format, we collected responses from at least 10 participants. After three to four participants had provided feedback on a given format, the study team reviewed the transcripts and field notes and modified selected formats based on this feedback. For example, several participants who reviewed the “Risk Level” format stated that the absence of the actual HbA1c value from the format was confusing, and therefore the format was modified to include the HbA1c value. All formats had one or two iterations. The number of participants who viewed the initial versions of each format, any changes made following initial feedback, and the number who viewed each revised version are included in Supplemental Table 3.

The interview transcripts were then analyzed using a thematic analysis approach (performed by two independent coders) and five key themes regarding format attributes that aided interpretation and improved clarity were identified: 1) the use of the colors red, yellow, and green together, 2) the presence of the actual HbA1c value in addition to pictorial representations of control, 3) consistent and intuitive directionality (low = better control), 4) inclusion of clearly identified goals/targets, and 5) the depiction of HbA1c change over time.

For most interviewees, the colors red, yellow, and green together aided interpretation. When paired with green, red was interpreted as high severity and susceptibility to diabetes-related complications.

*Green means you're good, it's go. Red means danger, that you need to watch out.*

*Yeah, and red is very poor. I mean it’s like stop. Stop whatever you’re doing here and this* [referring to yellow] *is a caution.*

Most of the reviewed formats did not include the numeric HbA1c value and consequently were felt to lack the specificity needed to understand one’s level of diabetes control. Many interviewees expressed that numbers provided more precise information about their diabetes severity.

*I want to see the number. I want to see exactly where I’m at, the numbers.*

*I’m wondering what the heck I’m looking at in the range without those numbers. Without the numbers it's kind of like just guesswork.*

All interviewees knew that the goal was to have their “numbers”, whether it was a finger stick blood glucose or the HbA1c, be lower rather than higher. This interpretation of low as indicating better control than high was one factor that caused confusion with formats where the “best” control level was located at the top of the page or on the right side of the page.

*Number three* [referring to life force format indicating “full” force], *because I’m looking at the level, now mind you, and again, I don’t know, but I’m looking at if something, I know when myself is way up, I ain’t feeling any too good … Anybody use that word up.*

Several interviewees suggested that inclusion of a clear goal or target HbA1c would be informative and motivating and felt that information about current status alone was insufficient.

*Because it* [referring to the time trend format] *actually has goal and whatnot … this one* [control level], *it only show the number. It don’t show you your goal or where you should be trying to strive for.*

Finally, a number of interviewees commented that information on their past diabetes control would be valuable. The ability to see improvement or worsening was viewed as useful, and for some, motivating.

*Without having something that goes over a distance, then you’re only stuck with what you got today. I think I really would like to see today, yesterday and the day before that. You can make a better comparison, I think.*

*I like how it shows where you were now, six months ago, and a year ago. It shows you if you’re growing, and that’s important.*

**Fig. 1:**
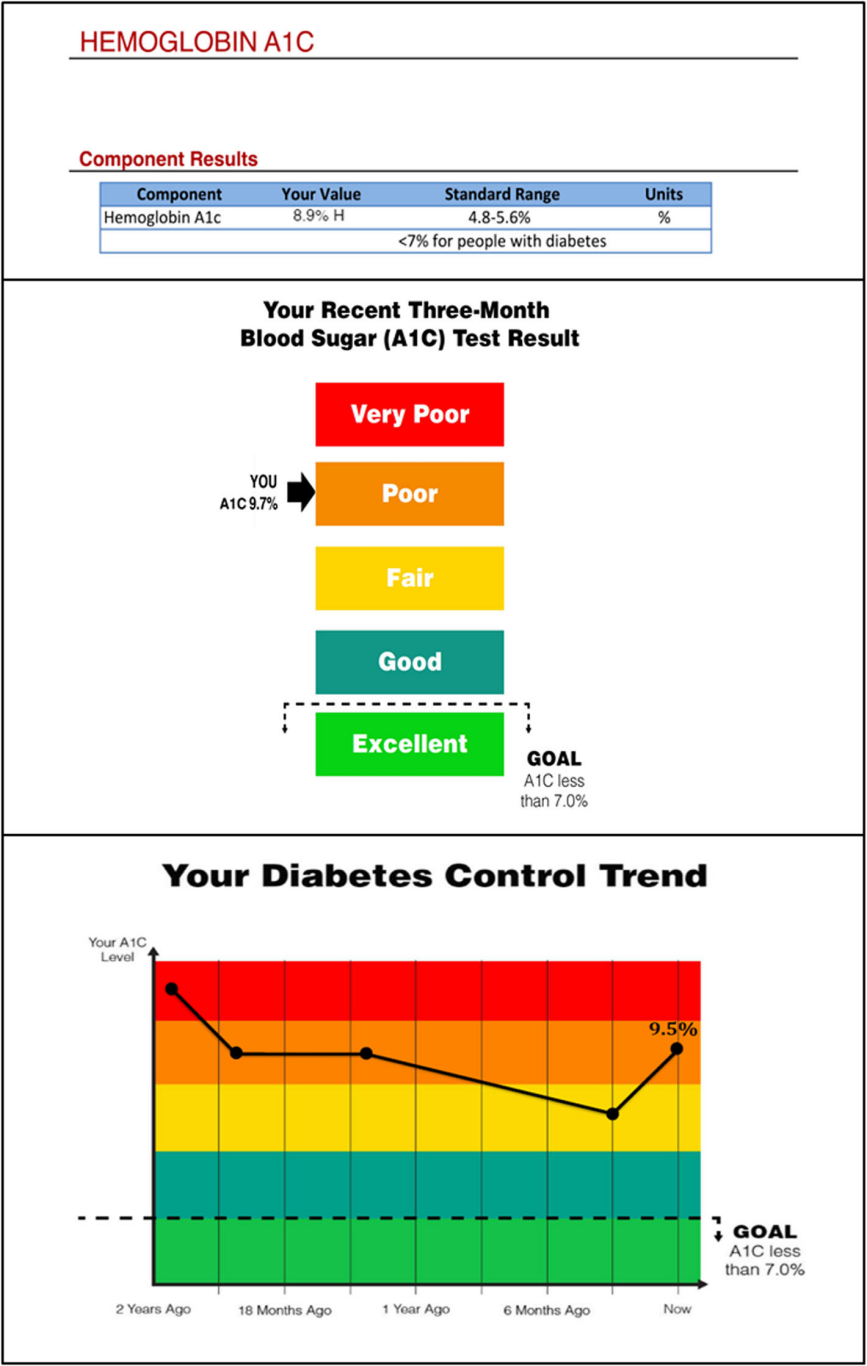
Formats for communicating the HbA1c value tested in each study arm. Top: Standard (control), Middle: Words format, Bottom: Graph format

These five identified attribute themes, along with representative quotes regarding specific formats, and the two quantitative measures informed the selection and design of the two formats tested in this RCT (Supplemental Table 2). The “Words” format and the “Graph” format were chosen as they incorporated most or all the attributes that interview participants felt aided format interpretation or enhanced format clarity (Fig. 1). The Words format reflected four of the five attribute themes: used green, yellow, red color scheme, included the numerical HbA1c value, had intuitive directionality (goal level at the bottom), and made the goal HbA1c clear. The choice of the Graph format was more difficult given that many had difficulty with interpetation of a graph during the interviews. However, the Graph format allowed for the depiction of HbA1c change over time. To increase the Graph format’s clarity, a green-to-red color scale was overlaid on the plot area. With this addition, the final Graph format exhibited all five of the attribute themes: the green, yellow, red color combination, appearance of the actual HbA1c value, intuitive directionality (goal level is at the bottom), presence of a clear target, and depiction of change in level of control over time.

Blank versions of the Words and Graph formats were sent to 15 primary care providers and five endocrinologists to collect their input on the HbA1c ranges corresponding to the five levels on each format (i.e., What HbA1c value range equals “excellent” control or Green? What HbA1c value range equals very “poor” control or Red?). The mean values of these clinicians’ responses were used to determine the HbA1c ranges on the two tested formats. Several clinicians felt that the HbA1c ranges they provided were not appropriate for older patients; based on this feedback, age ≤ 75 years was specified as an eligibility criterion for the subsequent RCT.

### Testing the designed formats

Eligible participants were randomized to receive individualized information (referred to as the report) about their current glycemic control in one of three ways (Fig. 1): 1) a standard lab report format (Control arm), 2) Words format, or 3) Graph format. Participants received two reports during the six-month intervention period. The first reports were mailed within 2 weeks of study enrollment and receipt was confirmed by phone. The first reports reflected participants’ HbA1c values at the time of study enrollment. Based on the recommended frequency of HbA1c testing for individuals not meeting glycemic targets, the second report was mailed 3 months following enrollment. This three-month report reflected any updated HbA1c results. If no new value was available, the value from the first report was used. During the study, it was assumed that participants continued to receive routine care and information regarding their diabetes management from their primary care and other providers.

#### Study population

Participants were recruited from four academically affiliated primary care practices (three urban and one suburban). Primary care providers were asked permission to enroll their eligible patients. Potential participants were identified via the electronic health record (EHR) using the following criteria: age 18–75 years and at least two HbA1c values ≥8% during the prior 2 years (one from within 3 months of enrollment). Anyone who self-reported pregnancy or who did not speak sufficient English to provide informed consent was excluded.

#### Randomization

Once eligibility was determined, verbal consent was obtained and the pre-intervention survey was administered, participants were randomized to one of the three study arms (simple randomization, with equal chance of assignment to each arm) by selection of a randomly ordered, sealed envelope. A biostatistician generated the randomization scheme and a research assistant (not the one performing enrollment) assembled the envelopes, ensuring that the allocation was concealed from the enrolling research assistant until after each individual was enrolled in the study. Given the nature of the interventions, blinding of the participants and research team was not feasible.

#### Study outcomes

The primary outcome was change in HbA1c at 6 months following enrollment. Secondary outcomes included changes in participants’ responses to five pre- and post-intervention survey questions and their choice of participation incentive. The five survey questions were adapted from a previously used instrument utilized to assess domains of the HBM in adults with diabetes and included: 1) perceived risk of diabetes-related complications, 2) perceived risk of a shortened life span due to diabetes, 3) perceived seriousness of diabetes, 4) assessment of current diabetes control, and 5) report of having a diabetes management goal [22]. Responses to these five questions are collectively referred to as “diabetes-related perceptions”. To measure differences in participants’ responses following the intervention, we allowed participants to choose their first participation incentive (i.e., to assess whether receiving information on current sub-optimal glycemic control in different formats prompted different incentive choices). Participants were given the choice of six options, each valued at approximately $20. Given the emphasis on diet and nutrition in ideal diabetes self-care, the incentive choices were dichotomized into food and non-food options. The food options included gifts cards to McDonald’s, Subway, Domino’s Pizza, or Dunkin’ Donuts. The non-food options included an electronic pedometer, a water bottle and exercise towel, or a CVS gift card. The retailers were chosen based on their locations in the neighborhoods where participants were most likely to reside. While the choice of any of incentive did not guarantee a more or less healthy choice (e.g., people may use their food gift cards to buy black coffee or a salad, or use their CVS gift card for candy), we wanted to see if receiving information on current glycemic control in different ways influenced participants’ choices.

#### Data collection

A telephone survey conducted at enrollment collected answers to the five diabetes-related perception questions, as well as information on participant demographics, diabetes history (e.g., duration, related complications), and the Whooley two-item depression screening tool [23]. Validated scales were used to assess participants’ diabetes self-efficacy, diabetes-related locus of control (internal, external, or chance), and health numeracy (measured by the subjective numeracy scale) [22, 24, 25].

Following receipt of their initial diabetes reports (two to three weeks following enrollment), a shorter post-intervention telephone survey was administered. This survey included the five diabetes-related perception questions. Following completion of this post-intervention survey, participants chose their first participation incentive (choice of the six options valued at $20).

At six months following enrollment, the EHR was queried for six-month HbA1c values collected through routine care. If no result was available, we arranged for participants to come to the research office. Once a six-month value was available, participants received the second participation incentive ($40 CVS gift card).

#### Statistical analysis

The sample size was chosen to provide at least 80% power to detect a difference of 0.8% in the primary outcome of change in HbA1c values at six-months, while also providing 80% power to detect a 25% between-arm difference in the secondary outcome of participation incentive choice. An effect size of 0.8% was chosen as HbA1c differences of this size have been shown to be associated with clinical benefits, and the magnitude was less than would be expected with more intense interventions [26, 27]. For the primary outcome, using an alpha value of 0.017 to allow for pairwise comparisons between all three arms, we estimated a required sample size of 74 participants per arm, for a total of 222. For the dichotomous outcome, using an alpha value of 0.025 to allow for comparisons between each experimental arm with the control arm, a sample size of 78 per arm was required, for a total of 234. The target enrollment was therefore set at 234.

For the primary outcome, we used multiple imputation to address missing six-month HbA1c values. The imputation models included the following participant characteristics (collected during the pre-intervention survey): race/ethnicity, age, gender, educational attainment, typical grades received in school, diabetes education, income, disease duration, insulin use, diabetes-related complications, perceived risk of complications, perceived seriousness of disease, depression, internal loci of control, and subjective numeracy score. After imputing the data, we used ANOVA to test for differences in HbA1c change between the groups in each imputed data set, and then combined the results using standard formulae [28].

For four of the five diabetes-related perception questions (all except report of having a diabetes management goal), the five-category Likert scale responses were collapsed into three categories: responses one and two, three, and four and five. Because all participants had baseline HbA1c values ≥8% (above the recommended goal for all adults < 75 years), we categorized responses at either end of the Likert scales as more accurate (e.g., higher perceived risk for future diabetes-related complications, diabetes perceived as very serious health problem, reported currently not managing diabetes well), and less accurate (e.g., lower perceived risk of future diabetes-related complications, diabetes perceived as non-serious problem, or reported currently managing diabetes well). Answers to the question regarding the presence of a diabetes management goal were dichotomized. To evaluate changes in these perceptions following the intervention, we first compared pre-survey responses to assess any between-group differences at baseline. We then examined changes in the proportions of more accurate responses pre- and post-intervention in each arm using chi-2 tests. Between-arm differences in participation incentive choice were compared using chi-2 tests.

All analyses were pre-specified and performed using R Core Team 2016 (Vienna, Austria). The study protocol was approved by the local Institutional Review Board. The funding sources played no role in study design, conduct, or reporting.

## Results

**Fig. 2:**
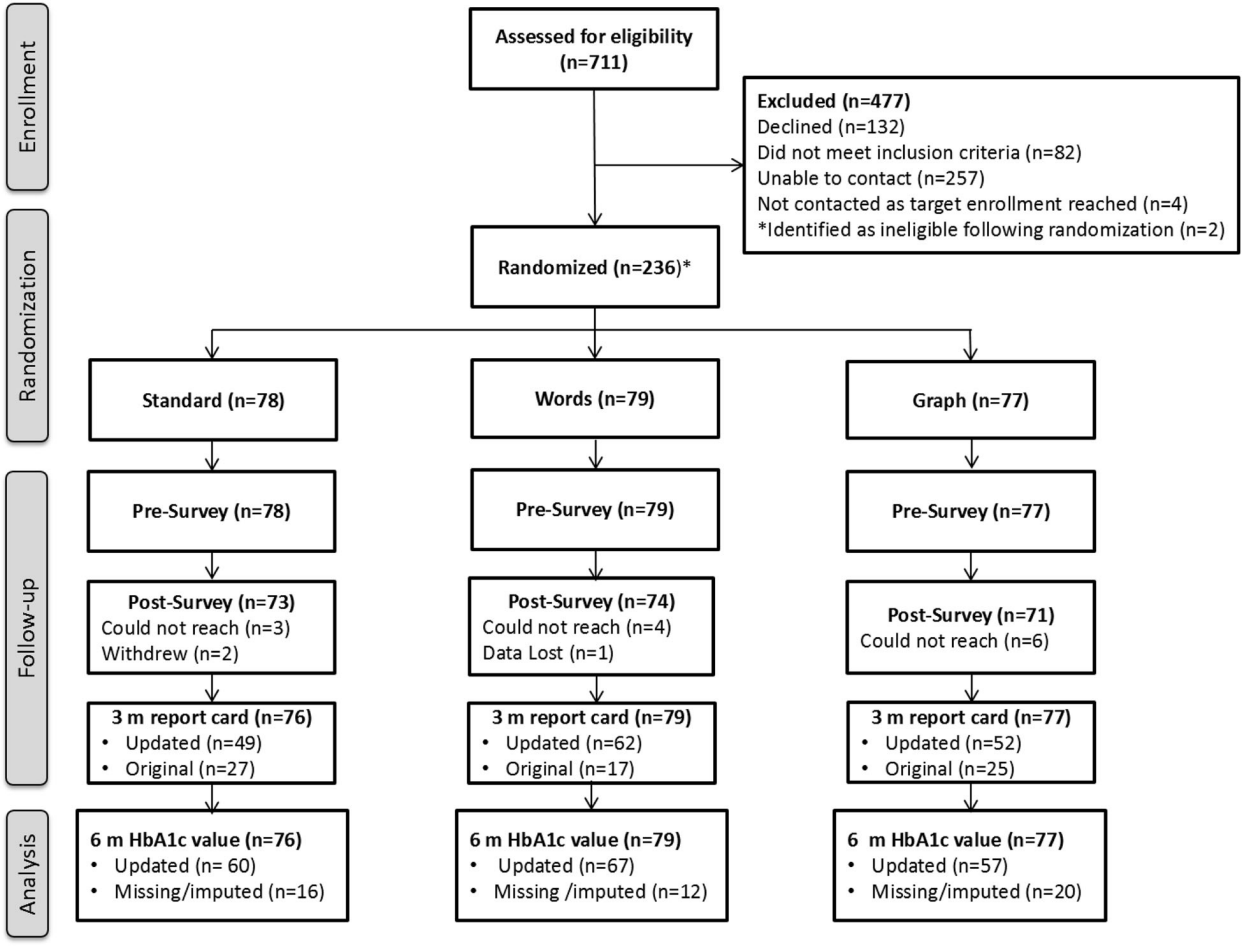
CONSORT Flow diagram overviewing randomization, enrollment, and follow-up

Between May and November 2014, 236 participants were enrolled in the study. A CONSORT flow diagram is provided in Fig. 2. The median HbA1c of enrolled participants was 9.1% (interquartile range 8.4–10.4%) and was comparable across study arms (Table 1). There were no notable differences among study arms in participants’ age, gender, race/ethnicity, duration of diabetes, or self-reported diabetes-related complications (Table 1).

**Table 1:** Participant characteristics by Arm

	Overall		Standard		Words		Graph	
	*n* = 234	100%	*n* = 78	33.3%	*n* = 79	33.8%	*n* = 77	32.9%
**Age (mean years [SD])**	56.3 (10.5)		57.8 (10.1)		56.1 (10.9)		54.9 (10.5)	
**Hispanic (%)**	4	1.7	1	1.3	1	1.3	2	2.6
**Race (%)**								
Black	180	76.9	59	75.6	63	79.7	58	71.4
White	42	17.9	14	17.9	14	17.7	14	18.2
Other	11	4.7	4	5.1	2	2.5	5	6.5
**Female (%)**	152	65.0	51	65.4	51	64.6	20	65.0
**Educational Attainment (%)**								
Less than high school	39	16.7	9	11.5	17	21.5	13	16.9
Completed high school	68	29.1	24	30.8	20	25.3	24	31.2
Some college or a technical degree	71	30.3	27	34.6	22	27.9	22	28.6
Bachelor’s/associates degree	34	14.5	9	11.5	12	15.2	13	16.9
Grad/professional degree	22	9.4	9	11.5	8	10.1	5	6.5
**Annual household income (%)**								
< $20,000	90	38.5	29	37.2	28	35.4	33	42.9
$20,000–$49,000	51	21.8	17	21.8	21	26.6	13	16.9
$50,000–$80,000	29	12.4	11	14.1	12	15.2	6	7.8
> $80,000	26	11.1	6	7.7	6	7.6	12	15.6
Missing	38	16.2	15	19.2	12	15.2	13	16.9
**Initial Hemoglobin A1c (Median % [IQR])**	9.1 (8.4–10.4)		9.2 (8.5–10.8)		9.0 (8.4–10.2)		9.0 (8.4–10.1)	
**Diabetes duration, (Mean years [SD])**	15.1 (11.0)		14.5 (10.6)		13.7 (10.9)		17.3 (11.4)	
**On insulin (%)**	166	70.9	50	64.1	58	73.4	58	75.3
**On an oral diabetes medication (%)**	148	63.2	57	73.1	48	60.8	43	55.8
**Self-reported diabetes-related complication (%)**	92	39.3	29	37.2	28	35.4	35	45.5
**Prior diabetes education class (%)**	117	50.0	34	43.6	42	53.2	41	53.2
**Self-reported hypertension (%)**	188	80.3	62	79.5	66	83.5	60	77.9
**Self-reported hyperlipidemia (%)**	172	73.5	54	69.2	56	70.9	62	80.5
**Positive depression screen (%)**^a^	102	43.6	36	46.2	31	39.2	35	45.5
**High diabetes self-efficacy (%)**^b^	125	53.4	43	55.1	47	59.5	35	45.5
**Diabetes locus of control (%)**^**c**^								
% Internal	149	63.7	54	69.2	52	65.8	43	55.8
% External	38	16.2	8	10.3	11	13.9	19	24.7
% Chance	8	3.4	2	2.6	3	3.8	3	3.9
**Subjective Numeracy Score (Mean [SD])**^**d**^	3.1 (1.1)		3.1 (1.3)		3.2 (1.0)		3.1 (1.0)	

*SD* standard deviation, *IQR* inter-quartile range

^a^Positive depression screen defined as a “Yes” response to ≥1 question

^b^High diabetes self-efficacy defined by a score above the population median

^c^Diabetes locus of control defined by the majority of each participant’s responses (e.g., if most responses were consistent with external loci of control, that participant was classified as having an external locus of control

^d^Subjective numeracy score measured on a scale from 1 (low numeracy) to 6 (high numeracy)

No significant differences in HbA1c change at 6 months were observed between arms (Table 2). There was no difference in the frequency of missing six-month HbA1c values between arms (20.5% Control vs. 15.2% Words vs. 26.0% Graph, *p* = 0.25); as described we used multiple imputation method to account for missing HbA1c values. A sensitivity analysis examining HbA1c change using only subjects with available six-month values (i.e., without imputation of the missing values) yielded results consistent with the primary analysis findings (*p* = 0.084 for between-arm comparisons).

**Table 2:** Change in HbA1c by study arm

Study Arm	N=	Baseline HbA1c Mean % (SD)	6-month HbA1c Mean % (SD)	Change Mean % (SD)	Mean between-arm difference in HbA1c change, % (95% confidence interval), ***p***-value	
					Words	Graph
Control	78	9.8 (1.7)	8.8 (1.7)	−1.04 (2.2)	0.45 (−0.22, 1.13), *p* = 0.18	0.50 (−0.18, 1.19), p = 0.15
Words	79	9.5 (1.6)	9.0 (1.7)	−0.59 (2.0)		0.05 (−0.64, 0.74), *p* = 0.89
Graph	77	9.6 (1.7)	9.1 (1.7)	−0.54 (2.1)		

Participants’ pre-intervention survey responses did not differ significantly by study group. Only a few participants in each arm did not consider diabetes to be a serious problem (11.5% Control vs. 16.5% Words vs. 9.1% Graph, *p* = 0.36) (Table 3). Many participants reported they were controlling their diabetes well (57.7% Control vs. 49.4% Words vs. 37.7% Graph, *p* = 0.04). The majority of participants reported a current diabetes management goal (91.0% Control vs. 82.3% Words vs. 90.9% Graph, *p* = 0.15) (Table 3).

**Table 3:** Changes in diabetes-related perceptions by study arm

	Control			Words			Graph		
	Pre-	Post-	p-value	Pre-	Post-	***p***-value	Pre-	Post-	p-value
**N=**	78	73		79	74		77	72	
Low risk for complications (%Yes)	47.4	49.3	1	43.0	39.2	0.66	32.5	40.3	0.38
Low likelihood of shortened life expectancy (%Yes)	61.5	56.2	0.33	55.7	47.3	0.181	46.8	54.2	0.69
Considers diabetes a non-serious problem (%Yes)	11.5	12.3	1	16.5	8.1	**0.04**	9.1	13.9	0.58
Reports currently managing diabetes well (%Yes)	57.7	47.9	0.21	49.4	35.1	0.05	37.7	36.1	1
Has a current diabetes management goal (%Yes)	91.0	97.3	0.13	82.3	93.2	**0.013**	90.9	97.2	0.13

When examining differences in the pre- and post-intervention responses for participants in the Words arm, we observed statistically significant changes in the responses to two questions (perceived disease seriousness and having a diabetes management goal) (Table 3). A change in assessment of current control was also noted but did not meet statistical significance. These observed changes were in the expected direction, towards more “accurate” responses. No significant changes in perceptions were noted for participants in the Control or Graph arms. There were no significant between-arm differences observed in participation incentive choice (“non-food” choice: 63% Control vs. 62% Words vs. 66% Graph, *p* = 0.87).

## Discussion

In this RCT of adults with inadequately controlled diabetes, receiving information on their HbA1c values in one of two new patient-informed visual formats had no differential effect on participants’ HbA1c values compared to standard presentation of the value. Still, for participants in the Words arm there was an observed increase in the accuracy of participants’ perceptions of their diabetes seriousness and in the proportion of participants who reported having a current diabetes management goal.

There are several possible reasons why no between-arm differences in HbA1c change were observed. Unlike other successful non-pharmacologic interventions for diabetes management, such as peer mentoring or care management, in our study, participants only received the information twice without any additional reinforcement or resources [29, 30]. While the strengths of the tested interventions include their low cost and easy implementation, the “light touch” nature of the interventions also makes the lack of between-arm differences in HbA1c unsurprising. In the HBM, perceived disease seriousness and susceptibility are only two of the key domains influencing the likelihood of health-related behavior change [10]. To trigger behavior change, the psychosocial barriers experienced by patients must also be addressed and cues to action from trusted providers may also be needed. In this study, participants were not provided with information on how to improve their diabetes control. Further, the interventions did not involve patients’ healthcare providers. However, this may be more reflective of the current environment where many patients review their lab results independently using online portals. Still, this type of communication intervention may be more potent if linked with provider and/or case manager support to identify existing barriers to management or paired with personalized action planning and goal setting. Additionally, the lack of observed effect may reflect the inherent nature of the HbA1c value itself. It may be hard for people to relate their day-to-day health behaviors to a lab result they receive at most every 3 months. The tested formats may have been more helpful if linked with more proximal measures of blood sugar management (e.g., percent of weekly fingerstick readings in target range).

The increased accuracy of perceived diabetes seriousness for participants in the Words arm suggests that our approach can improve communication regarding glycemic control for some patients with diabetes. However, the high percentage of enrolled participants (in all arms) who reported controlling their diabetes well (in spite of a median baseline HbA1c of 9.1%) and the absence of change in participants’ assessments of their level of control following the intervention, raise questions about what information patients are using to gauge their diabetes control. We assumed that patients’ diabetes-related perceptions were influenced mainly by the HbA1c value; however, patients may assess their diabetes using a different rubric (e.g., symptom severity, number of medications taken), therefore diminishing the relevance of the tested interventions [31].

The study findings must be interpreted in the context of some important limitations. All participants were patients at a small group of practices, and the majority were Black, potentially limiting generalizability of the findings to other populations. The effect size used to determine the sample size may have been too large given the low intensity of the tested interventions. We were not able to test the interventions in the real-world context (i.e., delay between HbA1c test and report mailing and the formats were not integrated on the online portal). We assessed between-arm differences in participation incentive choice; however, we did not capture the purchases made with the chosen incentive, and the low income level of many participants could have influenced the choice between food and non-food incentives. Though we asked participants about their diabetes-related perceptions, we did not assess other key factors such as knowledge, activation level, and medication adherence. The use of red, yellow, and green together was informed by the interviewees; still, this color combination may have provoked fear in recipients (e.g., forest fire risk scale). And, while fear-based messages can be motivating, they may not be ideally suited for promoting positive diabetes-related behavior changes [32]. First, there may be limits to the level of fear that is motivating, and past work has demonstrated many individuals already fear disease-related complications and actually overestimate their risk for these outcomes [33–36]. Also, for fear to effectively shift behaviors, individuals need to know what actions to take to prevent the feared outcomes, information that was not included with the tested formats [33]. Finally, we cannot differentiate whether participants had type 1 or type 2 diabetes; the proportion (63.2%) taking oral diabetes medications suggests most would be classified as having type 2 diabetes.

## Conclusions

In conclusion, the tested formats did not significantly improve participants’ glycemic control However, findings suggest that one new format improved the accuracy of participants’ perceived severity of disease and the proportion who reported having a disease management goal. There is value in trying to improve the effectiveness and patient-centeredness of our communication with patients with diabetes. To start, all patients deserve to have a basic understanding of their health and healthcare. Further, better patient ratings of provider communication are associated with higher patient satisfaction and medication adherence [37, 38]. Finally, rethinking our communication strategies is necessary to adapt to the changing communication landscape of healthcare. As patients independently access and review their medical data on EHR-based portals, communication approaches that make the interpretation of important lab results, like the HbA1c, easier for patients are essential to the care experience, even if they do not measurably impact traditional disease-related care outcomes and metrics.

## Supplementary information

**Additional file 1: Supplemental Figure 1** Figure showing the formats that were reviewed by interviewees to inform design of the tested formats, Words and Graph.

**Additional file 2 Supplemental Table 1** Interviewee Characteristics. **Supplemental Table 2** Format description, representative quotes, and results of ranking accuracy and clarity rating from patient interviews during intervention development. **Supplemental Table 3** Number of participants who reviewed each format and changes made to formats based on initial feedback.

## Data Availability

The datasets generated and/or analyzed during the current study are not publicly available due to institutional policies but are available from the corresponding author on reasonable request and with the appropriate IRB approvals.

## Abbreviations

EHR: Electronic health record
HbA1c: Hemoglobin A1c
HBM: Health belief model
