# Supplementary figures and images for: Testing patient-informed approaches for visually depicting the hemoglobin A1c value to patients with poorly controlled diabetes: a randomized, controlled trial
Source: BMC Health Serv Res. 2020 Mar 6;20:178. doi: 10.1186/s12913-020-5035-8 (PMC7059706; doi:10.1186/s12913-020-5035-8)

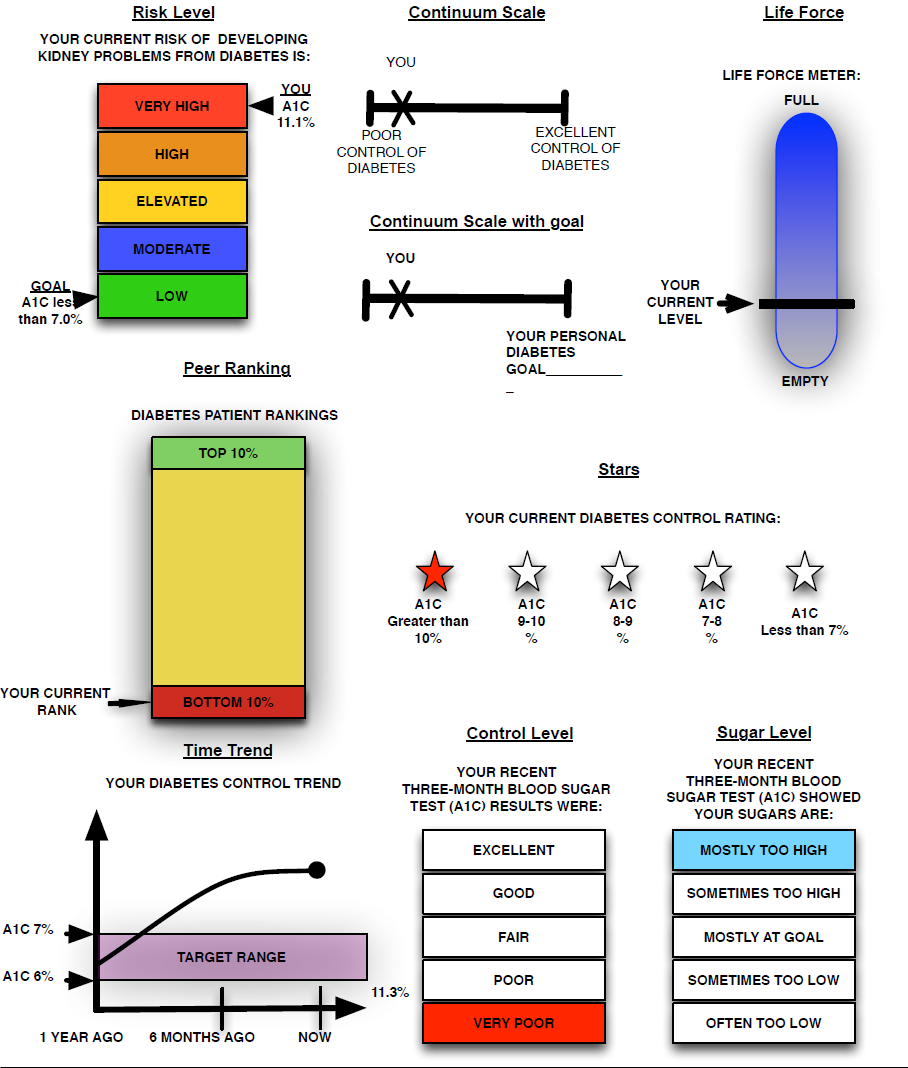

Supplement: Supplementary file 1 — Additional file 1: Supplemental Figure 1 Figure showing the formats that were reviewed by interviewees to inform design of the tested formats, Words and Graph. [file 12913_2020_5035_MOESM1_ESM.png]
