# Supplementary material for: Testing patient-informed approaches for visually depicting the hemoglobin A1c value to patients with poorly controlled diabetes: a randomized, controlled trial
Source: BMC Health Serv Res. 2020 Mar 6;20:178. doi: 10.1186/s12913-020-5035-8 (PMC7059706; doi:10.1186/s12913-020-5035-8)
Supplement: Supplementary file 2 — Additional file 2 Supplemental Table 1 Interviewee Characteristics. Supplemental Table 2 Format description, representative quotes, and results of ranking accuracy and clarity rating from patient interviews during intervention development. Supplemental Table 3 Number of participants who reviewed each format and changes made to formats based on initial feedback. [file 12913_2020_5035_MOESM2_ESM.docx]

| **Supplemental Table 1. Interviewee Characteristics** | | |
| --- | --- | --- |
| **Characteristic** | **N=25** | **%** |
| Age (mean years ± SD^a^) |  | 57±13 |
| Gender |  |  |
| Female | 17 | 68 |
| Ethnicity |  |  |
| Hispanic | 1 | 4 |
| Race |  |  |
| Black | 21 | 84 |
| White | 1 | 4 |
| Multiple^b^ | 3 | 12 |
| Education |  |  |
| Less than High School | 3 | 12 |
| High School or GED^c^ | 13 | 52 |
| Some College/Technical School | 6 | 24 |
| College or beyond | 3 | 12 |
| Years since diabetes diagnosis (mean ± SD) |  | 11±7 |
| Experienced a diabetes-related complication (Yes) | 9 | 36 |
| Diabetes Treatment |  |  |
| Oral medications only | 12 | 48 |
| Insulin | 6 | 24 |
| Oral medications & Insulin | 4 | 16 |
| Diet only | 3 | 12 |
| *^a^SD=standard deviation.*  *^b^*Multiple= Individual 1: Black, White, and Asian, Individual 2: Black and Native American, Individual 3: Black and White  ^c^GED= General equivalency diploma | | |

|  | **Supplemental Table 2. Format description, representative quotes, and results of ranking accuracy and clarity rating from patient interviews during intervention development** | | | |
| --- | --- | --- | --- | --- |
| **Format** | **Format description** | **Representative quotes** | **Ranked accurately^a^** | **Rated clarity highly^b^** |
| **Risk Level** | Color-based scale describing risk of adverse outcome [[7-9](#_ENREF_7)]. Example: forest fire risk | “All I got to do is see the color, and then I would exactly know look, it’s too high.”  “Interviewer: Which one would you say is the best…? Participant: Without looking I will say green. Interviewer: And which one is the worst? Participant: Of course, the red.” | 90% | 90% |
| **Continuum Scale** | Non-numeric scale showing progress towards goal[[11](#_ENREF_11)]. Example: fundraising progress tracker | “It's not adult friendly. It's just…a picture, not even number or information or your information. It doesn't even tell you where you're at. It just says you with no numbers. “  “Come on, that’s a patient, I don’t think – I wouldn’t want to see none of this, you would insult my intelligence if you brought something like this in front of me.” | 90% | 70% |
| **Continuum Scale with goal** | More personalized version of continuum scale[[11](#_ENREF_11)]. Example: fundraising progress tracker | “Just guessing, because it’s no numbers or nothing, so I don't know if I'm going up or going down.”  “I mean, your doctor is your physician. He can have goals for you, but you supposed to have your own goals, where you want to be, you’re the patient.” | 11% | 30% |
| **Life Force** | Shows movement away or towards goal[[10](#_ENREF_10), [11](#_ENREF_11)]. Example: video game life meter | “What the hell is a life force meter?”  “If full means you're in full – you know, your diabetes is fully happening.”  “how can a doctor detect just how happy you are, you know what I mean? How in the hell do they know?” | 36% | 30% |
| **Peer Ranking** | Comparison to peers [[13](#_ENREF_13)]. Example: School class ranking | “This is the best one, and the reason I’m saying this is because the current ranking is in the green, and green means good or excellent”  “I don’t know if it’s the bottom is the bad or the top is…”  “[I]f you're the top ten percent, you're taking medicine three times a day, and it's usually insulin in a shot.” | 40% | 40% |
| **Stars** | Non-numeric scale commonly used in other settings[[15](#_ENREF_15)]. Example: Yelp, iTunes | “[I]t’s a tad bit confusing right now. So I don’t have like a key or anything to go by. I don't know what the white stars mean, I don't know what the red stars mean.”  “They’re starting from the left to the right. They’re starting low, less than – okay. This is good. This is high and I think you’re a little bit messed up. I don’t understand these stars that much.” | 70% | 30% |
| **Time Trend** | Depicts changes over time, used to track other health information[[12](#_ENREF_12)]. Example: Weight trackers | “for somebody that didn’t really enjoy math and looking at grids, I don’t think this would be a good method.”  “Well, the graph actually breaks it out, and it leaves you with less questions with just a number. Because some people like diagrams to see their progression.” | 58% | 30% |
| **Control Level** | Describes without jargon, uses color-based scale[[7](#_ENREF_7), [14](#_ENREF_14)]. Example: Traffic light colors, cholesterol (LDL=” bad” cholesterol) | “Because it tells you right there, excellent and very poor…this might be better with the colors on it for an elderly person. And the words are written nice and large.” | 100% | 80% |
| **Sugar Level** | Equates blood sugar to potentially more familiar measure, minimizes medical jargon[[14](#_ENREF_14)]. Example: Cholesterol (LDL=” bad” cholesterol | “Because it’s telling you exactly what's going on. You get your test back and saying, ‘Oh, your sugars are too low.”  “See, I don't know too much about when your sugar level is too low…you always hear people talk about their sugar level being too high.”  “It's too much. It's too much. I don't need all this sugars are mostly too high. I don't need all those words.” | 100% | 30% |
| ^a^Accurate ranking was defined as a binary variable with “Accurate” defined as correct identification of the versions of the format depicting the “best” control and the “worst” control  ^b^High clarity rating was defined as those who gave a clarity rating of “4” or “5” on a 5-point Likert scale | | | | |

| **Supplemental Table 3. Number of participants who reviewed each format and changes made to formats based on initial feedback** | | | |
| --- | --- | --- | --- |
| **Format** | **# who viewed initial version** | **Changes made based on initial feedback** | **# who viewed revised version** |
| **Risk Level** | 4 | Added HbA1c value and HbA1c goal | 6 |
| **Continuum Scale** | 10 | None | Not applicable |
| **Continuum Scale with goal** | 10 | None | Not applicable |
| **Life Force** | 10 | None | Not applicable |
| **Peer Ranking** | 10 | None | Not applicable |
| **Stars** | 3 | Added red color to “filled-in” stars and added HbA1c ranges | 7 |
| **Time Trend** | 3 | Moved target region to sit on x-axis and added HbA1C value and HbA1c goal range | 7 |
| **Control Level** | 3 | Added HbA1c value | 7 |
| **Sugar Level** | 3 | Shortened labels | 7 |
